# The Cement Problem

**Published:** 1905-09-15

**Authors:** G. C. Poundstone

**Affiliations:** Chicago


### THE CEMENT PROBLEM.

BY G. C. POUNDSTONE, D.D.S., OF CHICAGO.

The July *Dental Cosmos* contains two articles on scientific investigation of the cement characteristics as applied to inlay work. Dr. Poundstone endeavors to determine the comparative values of ten different cements, which he purchased in the open market, making his comparisons on the following basis of requirement for setting porcelain inlays. —First, capacity of being compressed into a very thin film, at least as thin as the metallic matrix used in baking the inlay. Second, comparative adhesive qualities. Third, comparative expansion or contraction. Fourth, capacity for absorbing moisture.

The first series of experiments were designed to show the possibilities for compression into thin films. A batch of each cement was mixed with glycerine to prevent chemical action, and spread as thinly as possible on glass slides for microscopic examination. The object was to ascertain the form and size of the constituent powder granules. The microscope revealed the fact that there was considerable variation in the granules, from very fine powder to crystals of considerable size. By classifying the granules into "powder," "medium granules" and "large granules," it was found that the amounts of these three classes varied in the different cements examined, and probably modified their use for inlay purposes accordingly.

To avoid unpleasant complications, the different cements were designated by a letter instead of their trade names. This will sufficiently identify the cements for the purpose of this experiment.

| Cement | LARGE GRANULES | | MEDIUM GRANULES | | POWDER |
|---|---|---|---|---|---|
| | Relative Number | Size of Largest | Relative Number | Average Size | |
| A | Few | 35 microns | Many | 8–15 microns | Little |
| B | Few | 50 " | Few | 10–15 " | Much |
| C | Few | 50 " | Many | 7–15 " | Little |
| D | Very few | 56 " | Few | 12–18 " | Very much |
| E | Very few | 25 " | Very many | 10–15 " | Little |
| F | Few | 56 " | Many | 16–30 " | Much |
| G | Few | 46 " | Many | 16–18 " | Much |
| H | Few | 40 " | Many | 5–20 " | Little |
| I | Very few | 27 " | Very many | 10–15 " | Little |
| J | Very few | 25 " | Very many | 8–15 | Much |

**NOTE.**—Twenty-five microns approximately measure the thickness of ordinary inlay platinum or one one-thousandth of an inch.

For the second series slides were made from the various powders, each mixed with its regular cement liquid in the manner indicated by the manufacturer, and to a consistence suitable for setting an inlay. The cover glasses were subjected to pressure to get as thin a film as possible. These slides were then studied with the microscope. The process of setting was carefully watched for several hours, afterwards at intervals of a few hours, until no further change could be detected.

Cement A: The fine powder united with the liquid almost immediately, the small granules began to disappear and to give off bubbles which gradually increased in size and number until the field was well covered with them at the end of an hour. In twenty-four hours the fine powder had all united with the liquid and the large granules were slightly reduced in size. In three or four days many of the small granules developed into beautiful radiating crystals. At the end of a week many bubbles have disappeared, leaving large spaces, the cement turning gray. In two weeks the largest granules are still visible and the open spaces have become irregular.

Cement B: The rod-like crystals seen in this powder in the glycerine mix have almost entirely disappeared within twenty minutes after mixing with the cement liquid, the other granules show little change; the entire slide presents a gray appearance. A number of bubbles soon appear which expand, and in two or three hours a clear area forms around each bubble, around the edges of which crystallization is apparent. In five hours there are large open spaces to be seen throughout the film. In twenty-four hours circular areas of crystallization are numerous near the borders of the clear spaces, and open spaces occupy at least one-half of the slide, and crystallization is complete.

Cement C: The transparent crystals seen in the glycerine mix of this cement dissolve readily in the cement liquid, but the other granules are little affected. In one hour the film is dark-gray and granular. Subsequent changes come slowly. In two or three days crystallization takes place in stellate form, resembling the jewel called a "sunburst." The outermost crystals are large and have definite angles, and by transmitted light there is considerable irridescence.

Cement D: Immediately after mixing and fixing on the slide, a large number of small bubbles appear from the fine powder; these increase in size and number and unite with each other until they cover almost one-third of the slide. After a week they assume irregular forms, and crystallization begins along their borders. In four weeks the entire field becomes crystallized and all granules disappear. The open spaces are fringed with crystals. The crystals have numerous spine-like projections.

Cement E: At first the fine powder dissolves readily and the granules group themselves into chains or irregular forms, leaving large areas of liquid. The granules gradually dissolve. Crystallization begins in two days and continues for two months. The crystals are needle shape and radiate from centers.

Cement F: The fine powder unites with the liquid readily forming small bubbles, which increase in size for two or three days. The large granules lose their sharp edges. In three or four days crystallization is apparent and is completed in about six weeks.

Cement G: This is a slow setting cement. The fine powder begins to dissolve in one hour and is completed in twenty-four hours. No considerable number of bubbles appear.

Cement H: A large number of bubbles appear soon after it is mixed. In twenty-four hours the powder is dissolved, but the granules are only slightly affected. The slide is gray in color and the bubbles are irregular in form.

Cement I: The fine powder unites readily with the liquid. The largest granules arrange themselves into groups or chains with liquid between as in Cement E. A large number of bubbles appear in the clearer portions, apparently coming from the particles of fine powder. By the end of the week there is a large amount of crystallization. The bubbles change to large irregular spaces and the slide to a grayish and matted color.

Cement J: The fine powder dissolves quickly and a few bubbles appear in the first half hour. In two hours most of the granules have disappeared and crystallization has advanced to a considerable degree. Two forms of crystals appear; a rod or prism, square at the ends, and a rhombus with obtuse angles rounded. There is no definite arrangement of the crystals with reference to each other. In twenty-four hours the rounded angles of the large crystals become straight lines, making six-sided prisms.

Slides of the different cements were made in the same way and kept in water at body temperature, and studied in the same manner as above, and with substantially similar results, except that crystallization was hastened.

From these experiments it will be evident that cements containing large insoluble granules, are not suitable for inlays unless the matrix is materially thickened. If they should present themselves edgewise instead of flatty, a close joint would be impracticable.

Another set of experiments was made to determine the exact thickness of these granules when set.

Two cover glasses were accurately measured in the micrometer and a mix of cement of proper consistence for setting an inlay was made and placed between them. They were then subjected to a pressure of twenty-five pounds for fifteen minutes. This is the maximum pressure that would ever be brought by the fingers upon an inlay and is far beyond that which most operators would use, careful tests proving that from ten to fifteen pounds is all that can be exerted generally—and that for not more than two or three minutes at a time.

At the end of fifteen minutes the cover glasses with the film of cement between them were measured. They were again measured in twenty-four hours, and again in seventy-two hours, with the following results:

TESTS FOR EXPANSION OF COVER-GLASS FILMS. (DRY.)

| Cement | Thicknes of Cement after setting 15 min under 25 lbs pressure. | Thickness after setting 24 hours | Expansion | Subsequent Change |
|---|---|---|---|---|
| A | 21 microns | 21 microns | 0 microns | Slight expansion |
| B | 23 " | 43 " | 20 " | None |
| C | 22 " | 44 " | 22 " | None |
| D | 24 " | 25 " | 1 " | Slight increase in expansion |
| E | 27 " | 27 " | 0 " | None |
| F | 30 " | 34 " | 4 " | None |
| G | 32 " | 42 " | 10 " | None |
| H | 26 " | 27 " | 1 " | Slight increase in expansion |
| I | 31 " | 35 " | 4 " | Slight increase in expansion |
| J | 25 " | 33 " | 8 " | Slight increase in expansion |

Twenty-five microns is approximately 1-1000 of an inch. It would therefore seem that only five of the cements tested would permit the inlay to be placed into proper position if a 1-1000 matrix were used, and this would be under twenty-five pounds pressure. If this is true, then what will be the result in setting an inlay made to exactly fit the cavity by the swaging process? This: The inlay if flat will remain just the thickness of the cement film out of position, or if it be one with parallel or slightly converging sides, the larger granules of the cement will be forced to the botton of the cavity underneath the inlay and prevent it from being forced to position, while around the sides will be only the thinner portion of the cement which, owing to the excess of liquid, will be more readily soluble in the fluids of the mouth.

The expansion of these thin films is also worthy of careful consideration. For example: In *B*, *C* and *G*, in which the expansion is from 30 to 100 percent, we have an explanation for the broad band of cement showing around the inlay after a few days when at the time of setting the joint appeared to be perfect. *J* also expands too much, while *F* and *I* could be better. *A* and *E* show no expansion, but *A* is filled with bubbles, and it will be shown later that *E* lacks in adhesiveness. *D* contains far too many bubbles, and *H* has troubles of its own.

For the purpose of determining the adhesiveness of the different cements, tests were made as follows: Blocks of ivory were carefully prepared with the surfaces to be cemented together having an area of sixty square millimeters, approximately that of the surface of a large inlay. These surfaces were roughened with a vulcanite file and the blocks were then cemented together. They were kept dry for twenty-four hours, when they were pulled apart with the following results, the number of pounds given being the average of a number of trials in each case. The highest and the lowest tests are also given:

TESTS FOR ADHESIVENESS. (IVORY BLOCKS 60 SQ. MM.)

| Cement | Force necessary to separate after twenty-four hours | | |
|---|---|---|---|
| | Average Force | Highest | Lowest |
| A | 46½ lb. | 68 lb. | 28 lb. |
| B | 46 " | 60 " | 26 " |
| C | 44¾ " | 63 " | 32 " |
| D | 23½ " | 30 " | 18 " |
| E | 31¾ " | 37 " | 20 " |
| F | 45½ " | 70 " | 29 " |
| G | 59¼ " | 72 " | 51 " |
| H | 50¾ " | 68 " | 33 " |
| I | 42¼ " | 48 " | 28 " |
| I | 35¼ " | 42 " | 30 " |

Repeated tests were made by cementing the blocks together and keeping them in saliva in the incubator at thirty-seven degrees C., but in every instance the force necessary to separate them was so light that it was impossible to measure it with any degree of accuracy. These were flat surfaces, with the force applied perpendicular to the surface. When force was applied parallel with the surface, *i. e.*, when an attempt was made to slide the blocks apart, considerable resistance was met with, the film of cement apparently interlocking into the uneven surfaces of the blocks on either side.

In the tests the capillary attraction between the cement and the ivory block is greater than the adhesiveness of the cement, and this has been the case with all of my tests except those in which the cement was covered with varnish, which would theoretically make it the same as if kept dry. But in the mouth we have all met with cases of old cement fillings in which it was necessary to chip away the last vestige of filling from the walls of the cavity, the adhesion being perfect, thus proving that adhesion does, in some cases at least, take place.

It is my belief that most inlays are retained in position solely by the retention form of the cavity, the accurate fit of the inlay and the cement on the one hand, and the cavity wall and the cement on the other; and that after a few days there is a film of moisture between the cement and the cavity wall and between the cement and the inlay, the cement acting merely as a key in locking the inlay into the cavity by means of its projections extending into the irregularities of the cavity wall on the one hand, and the depressions in the inlay formed by etching or grinding on the other.

Cements that contain bubbles and open spaces will undoubtedly permit the penetration of moisture; but to more certainly demonstrate this another series of experiments was made. Cover glasses were carefully etched and cleansed, and a mix of cement placed between each pair. These were held firmly together until they had set, when they were placed in water-eosin and kept in the incubator at thirty-seven degrees C. They were examined from day to day and drawings were made showing the extent to which the eosin had penetrated.

This penetration of the eosin is generally not within the body of the cement itself, but is between the cement and the glass, and a number of those cements which permit the eosin to pass between themselves and the glass are the so-called impervious-to-moisture cements; *i. e.*, they will withstand the common tests of rolling up a pellet of the cement and dropping it into red ink or anilin dye, and after immersion for days, weeks or months show no signs of penetration of the coloring matter. But that test is of little or no value whatever, for the defect is not in the cement itself, but in the joint between the cement and the tooth. The cement may be perfectly insoluble, but that will not prevent moisture passing between it and the tooth. If moisture can enter, micro-organisms will enter, and decay result. This is further proved by the fact that nearly all old cement fillings that are removed from the cavities in the teeth are discolored completely over the surface that was in contact with the tooth, while in the center they are still as bright and clear as when first inserted.

The result of the foregoing observations and experiments may not offer much assistance to the dentist who is looking for the best cement; for it is difficult, if not impossible, to say which is the best, so much depending upon the particular purpose for which it is to be used. The individual case will, in a large measure, have to determine the kind of cement that will best fulfill its requirements. Again, so much depends upon proper mixing and care in keeping some cements that it is impossible to foretell the results of any one of them in the hands of different operators.

It is therefore advisable that every dentist shall do some experimenting that he may know from experience what is actually taking place as a result of his own manipulation. In so doing he may be able to better his own work, or at least, save himself from embarrassment through ignorance of that which he is using.

